# Implementing a Homogeneous Psychotherapy Group for Bipolar Disorder: Outcomes and Case Study

**DOI:** 10.1192/j.eurpsy.2026.11201

**Published:** 2026-07-31

**Authors:** M. Krevatin, L. Goršić, S. Kocijan, S. Caratan

**Affiliations:** 1 Day Hospital for non psychotic disorders, University Psychiatric Hospital Sveti Ivan, Zagreb; 2 Department of Psychiatry and Neurology, Faculty of Dental Medicine and Health J.J. Strossmayer, Osijek, Croatia

## Abstract

**Introduction:**

Bipolar affective disorder (BAD) is a chronic mental illness characterized by mood instability, impaired insight, and high suicide risk. While pharmacotherapy remains essential, patients often face ongoing psychosocial challenges and stigma. Group psychotherapy offers a supportive setting to reduce isolation and foster coping, but BAD patients are often considered difficult group members due to impulsivity and mood fluctuations. This work presents clinical experience and a case report illustrating therapeutic outcomes .

**Objectives:**

To evaluate the feasibility, challenges, and benefits of implementing a homogeneous group psychotherapy format for patients with BAD and to illustrate therapeutic group dynamics through a clinical case.

**Methods:**

Since October 2018, a BAD-specific psychotherapy group met weekly to complement pharmacotherapy. Sessions focused on psychoeducation about bipolar disorder, treatment adherence, suicide risk monitoring, and group cohesion development. Attendance, drop-out rates, group interactions, and setting adaptations in pandemic circumstances (outdoor and online formats) were monitored.

**Case Presentation:**

A 35-year-old married man, father of two, working in entertainment, participated regularly. Despite economic stability, he struggled with life dissatisfaction, traumatic childhood experiences, marital communication problems, and gambling addiction. The group provided a safe space to share intimate issues, normalize symptoms, and receive peer support. Acceptance empowered him to articulate needs and seek specialized gambling addiction treatment. He described the group as a “good mother” and found participation transformative, viewing bipolar disorder as ultimately contributing to his personal growth.

**Results:**

The group faced high drop-out and slower cohesion due to poor insight and behavioral challenges. Nevertheless, core members sustained attendance, fostering mutual support, reducing stigma, and enhancing psychosocial functioning. Group therapy was notably more effective during depressive phases and helped prevent suicidal impulses.

**Conclusions:**

A homogeneous group psychotherapy setting for patients with bipolar affective disorder offers a unique, non-judgmental microcosm that facilitates self-reflection, emotional support, and psychoeducation. It reduces stigma and promotes personal development while serving as a bridge to supplementary treatments such as specialized addiction therapy. The presented case illustrates the transformative potential of group therapy, demonstrating how consistent participation can empower patients to articulate their needs, seek additional treatment, and experience meaningful personal growth.

**Disclosure of Interest:**

None Declared

